# Energy-flux control of the steady-state, creep, and dynamic slip modes of faults

**DOI:** 10.1038/s41598-019-46922-1

**Published:** 2019-07-23

**Authors:** Ze’ev Reches, Ximeng Zu, Brett M. Carpenter

**Affiliations:** School of Geosciences, University of Oklahoma, Norman, OK USA

**Keywords:** Tectonics, Seismology, Geophysics

## Abstract

Faults exhibit a gamut of slip styles from stable sliding and creep events to earthquakes. These slip styles are affected by the fault properties, e.g., weakening or strengthening, and the properties of the loading system. Here, we investigate the poorly understood effect of energy-flux to the fault that should equal or exceed the energy-dissipation-rate along the slipping fault. We explore the relationship between energy-flux and slip style in shear experiments along granite and diorite laboratory faults, during which the faults were subjected to controlled energy-flux, and responded spontaneously to it. The monitored evolution of slip-velocity, shear stress, and slip-distance revealed three slip styles that depend on the applied energy-flux: (1) steady-state slip; (2) spontaneous creep events of small displacement with negligible weakening; and (3) spontaneous, unstable events with slip-velocities up to 0.8 m/s, slip-distances up to 0.5 m, and stress-drops up to 1 MPa, which are comparable to observed values of moderate earthquakes. These slip styles are similar in character to those observed along natural faults. We further propose that the rate of energy flow from crustal blocks can control the slip velocity during earthquakes.

## Introduction

## Energy-Flux and Fault-Slip

**Figure 1:**
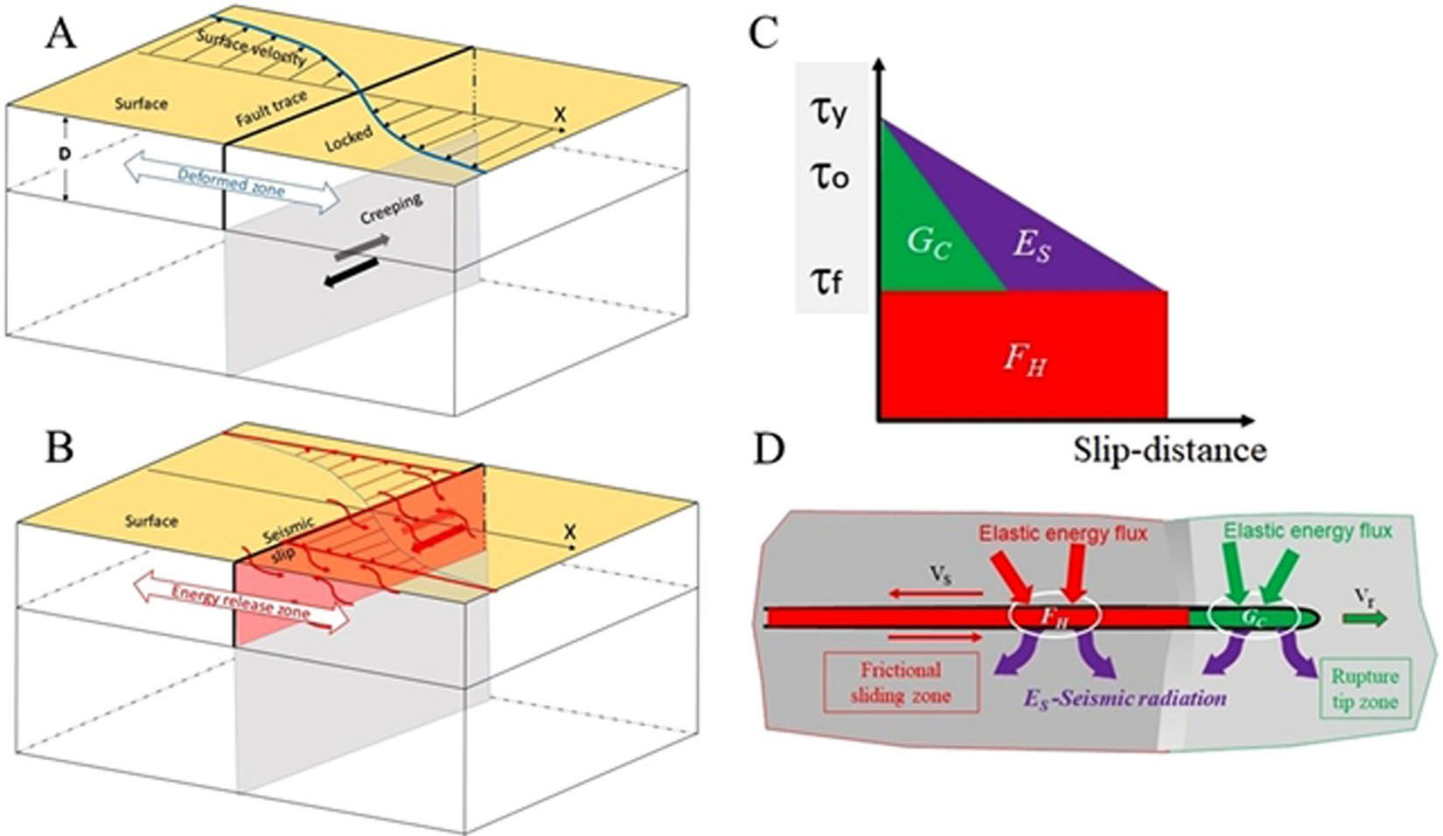
(**A**,**B**) Schematic view of an earthquake cycle along a large strike-slip fault. During the interseismic period (**A**), slip occurs along the deep part while elastically deforming (blue curve) the upper layer. During the earthquake (**B**), the locked fault slips unstably (pink zone) facilitated by energy-flux (red, wiggly arrows) from the energy release zone. (**C**) Energy dissipating processes^1,2^ during an earthquake as function of slip distance. G_C_–fracture energy; F_H_-frictional energy; E_S_- radiated, seismic energy. (**D**) Energy-flux toward the two general zones of energy dissipation: the rupture tip (green) and the frictional sliding block (red) in the wake of the earthquake rupture tip.

Earthquakes are fast slip events driven by the release of elastic energy that was accumulated in crustal blocks^1,2^. An example for this process is a large strike-slip fault (Fig. 1A) that during the interseismic period is locked in its upper part, while the deeper part slips stably and deforms the crustal blocks that bound the locked fault^3^. When the locked fault fails unstably, the earthquake slip releases the accumulated elastic energy (Fig. 1B). Even though the elastic energy is stored before the earthquake (Fig. 1A), its delivery is not instantaneous. To maintain slip, the energy-flux to the fault (Fig. 1B) should equal or exceed the energy-dissipation-rate along the fault. Thus, earthquake slip requires balancing two energy parameters: (A) a static balance between the total available elastic energy and the total energy dissipated along the fault; and (B) a dynamic balance between energy-flux to the fault and energy-dissipation-rate along the fault.

During an earthquake, the energy is dissipated by three main processes^4^ (Fig. 1C): fracture energy, G_C_, mostly at the rupture tip, frictional energy, F_H_, and seismically radiated energy, E_S_. A propagating earthquake may be divided into two general zones of energy dissipation (Fig. 1D). At the rupture tip, the dissipation is primarily by fracturing (G_C_) and radiation (E_S_)^4,5^ that together account for 10–20% of the total earthquake energy^1,4^. The rupture propagation velocity of a few km/s^6–9^ is limited by the energy-flux to the tip zone^9,10^. In the frictional sliding zone, which trails in the wake of the rupture tip (Fig. 1D), the energy is dissipated primarily by frictional processes, which are estimated to consume 80–90% of the total earthquake energy^4^. The dissipated energy is supplied by the release of elastic energy stored in the bounding crustal blocks. To maintain slip, the energy-flux to the slipping fault should equal, or exceed, the rate of energy-dissipation. Consider, for example, a large strike-slip fault, like the San Andreas fault, for which the interseismic elastic energy is stored within a 50–100 km wide zone^3,10^ (Fig. 1A) A large earthquake needs to drain the available elastic energy from the entire energy storage zone (Fig. 1B), and it could take 10–15 s for energy to reach the fault (assuming an energy flow rate equal to the shear wave speed). This finite time could bound the slip-velocity if the frictional dissipation rate exceeds the energy-flux.

Further, energy flux may control the slip style of a fault. The slip style may vary along a fault-zone with position or time, ranging from stable, steady-state creep to unstable rupture in earthquakes. For example, slip along segments of a large subduction zone may occur in four or five slip styles, including steady-slip, creep events and mega-earthquakes^11^. The active slip style on a given segment can be attributed to the composition of the gouge zone (e.g., talc, calcite, clay), environmental conditions (e.g., normal stress, temperature, pore pressure, loading style, and system stiffness)^12–15^. We argue here that the activation of a given style may also be affected by the rate of energy flow (energy-flux) to the fault segment.

The present analysis focuses on the effects of energy-flux on the slip style of experimental faults and possible application to natural earthquakes. The experiments are conducted at room conditions, low normal stresses, and the rotary shear is applied on cylindrical solid blocks composed of granite and diorite. Unlike typical friction experiments, in which the applied slip-velocity is controlled by the experimentalist^12,16–18^, in the present experiments, we select the intensity of the energy-flux and allow the fault to respond spontaneously. We envision that this loading style may apply to natural fault loading which is controlled by the energy supply rate from the host crustal blocks. The experimental observations reveal a spectrum of slip styles that depend of the experimental energy-flux. The stress-drops and slip-velocities associated with the observed slip events are comparable to other experiments and to earthquake observations.

## Experimental Setting

### Energy-flux and power-density control

Our central objective is to determine the effects of energy-flux on the frictional sliding of experimental faults (Methods). Controlling the energy-flux in experiments is difficult and a simple solution is to control the energy-dissipation-rate on the fault that by definition equals the energy-flux from the loading system. The energy-dissipation-rate per unit area, which is also defined as power-density^6,13^ is

Energy-flux = Energy-dissipation-rate per area = Power-density (PD), where

1 $${\rm{PD}}=[{\rm{slip}} \mbox{-} {\rm{velocity}}]\cdot [{\rm{shear}}\,{\rm{stress}}]={{\rm{V}}}_{{\rm{slip}}}\cdot \tau $$

with PD units of MW/m^2^ (for velocity, V_slip_, in m/s and shear stress, τ, in MPa). Our experimental system continuously monitors both slip-velocity and shear stress, and the loading motor velocity has a feedback control capacity on either torque or velocity. We developed an experimental procedure that uses these capacities to control the power-density (PD), and by doing so controls the energy-flux. The experimental fault directly controls the evolution of its frictional strength, which in turn controls the slip-velocity evolution by the programmed PD response of the apparatus loading system; both effects are without operator intervention.

We developed a dedicated LabView program that utilizes a ‘proportional–integral–derivative’ (PID) controller for operation and data acquisition under power-density control. Details of the control program are described in the Methods section. The PD control procedure is adjusted by tuning the PID parameters before running, and once the test starts, the interconnected system control and fault behavior are spontaneous (Methods). The accuracy of the control is limited by the sampling rate (256 Hz) of the algorithm and the apparatus motor PID sensitivity.

### Effective-power-density (PDE)

Two values of power-density are considered in the present analysis: the requested PD (equation 1 above), which is set by the operator, and the effective power-density, PDE, which is calculated from the monitored data,

$${\rm{PDE}}=[{\rm{measured}}\,{\rm{slip}} \mbox{-} {\rm{velocity}}]\cdot [{\rm{measured}}\,{\rm{shear}} \mbox{-} {\rm{stress}}]$$

**Figure 2:**
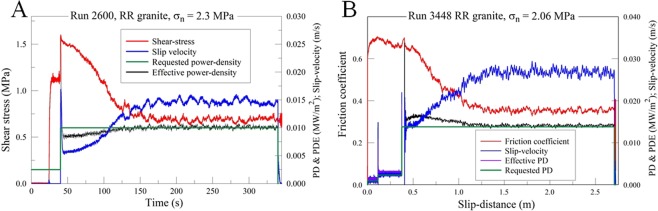
(**A**,**B**) The evolution of shear stress, slip-velocity, requested power-density (PD) and effective power-density (PDE) in stable-motion slip style for experiments #2600 (**A**) and #3448 (**B**).

We observed two basic styles of relationships between PDE (effective) and PD (requested) that we refer to as stable-motion and event-motion. In the stable-motion style, the effective PDE fits well the requested PD with overlapping curves of PD and PDE, e.g., experiments #2600 (Fig. 2A) and #3448 (Fig. 2B). The good fit fails temporarily during short periods of transition between PD levels, which are characterized by intense, temporal responses of friction and velocity due to limitation of the power control system. In the event-motion style, this fit disappears and the fault displays a sequences of multiple short slip events that are separated by periods of slow velocity or no-motion. These temporal events reflect the spontaneous interconnected response of the experimental fault and the loading system even under constant requested PD. These two styles of fault response produced three slip styles, steady-state, creep events, and stick-slips, that strongly depends the intensity of the requested PD, with additional contributions of the other controlling parameters (Methods). The experimental observations and the relationship between PD and PDE are described and analyzed below.

## Experimental Observations

### Stable-motion slip style

The evolution of the slip-velocity and shear-stress in the steady-state experiments is controlled by the requested power-density. Experiment #2600 with a granite sample was loaded under 2.3 MPa normal stress and then subjected to initial power-density loading of PD = 0.0025 MW/m^2^ that raised the shear stress to 1.2 MPa (Fig. 2A). This shear stress was insufficient to overcome the fault shear strength. When PD was raised to 0.01 MW/m^2^, the velocity quickly increased, the rising shear stress exceeded the static strength of 1.58 MPa, and fault slip initiated. The shear stress gradually dropped from the steady-state dynamic strength of 0.7 MPa (Fig. 2A). The control system increased the slip-velocity to a steady-state of 0.015 m/s, to match the requested PD. The effective power-density (black curve), agrees well with the requested PD (green curve, Fig. 2A) and the slip can continue for long distances at the steady-state value of energy-flux that maintains the frictional dissipation-rate.

In experiment #3448, with granite under 2.06 MPa normal stress, we used three nominal intensities of power-density, 0.00092, 0.0023, and 0.0138 MW/m^2^, and each intensity level was applied for a period of 100 s. Figure 2B displays the evolution of the slip-velocity and the friction coefficient as function of the slip-distance. A good fit is noted between the effective PDE (black curve) and requested PD (green curve, Fig. 2B) for the long periods of constant PD, yet, intense, temporal responses of friction and velocity occur during the transition between PD levels, due to speed limit of the power-control system (discussed above).

In these and in similar experiments, the loading system applied a few levels of power-density, and the slip-velocity developed spontaneously as function of the fault resistance to slip. The faults become weaker with progressive slip, similarly to classical, constant velocity experiments^12,16–18^. However, unlike the constant velocity experiments, here, the slip-velocity is increased by the power control system as is demanded by the weakening fault (Fig. 2A,B).

### Event-motion slip style

#### Requested and effective power-density

**Figure 3:**
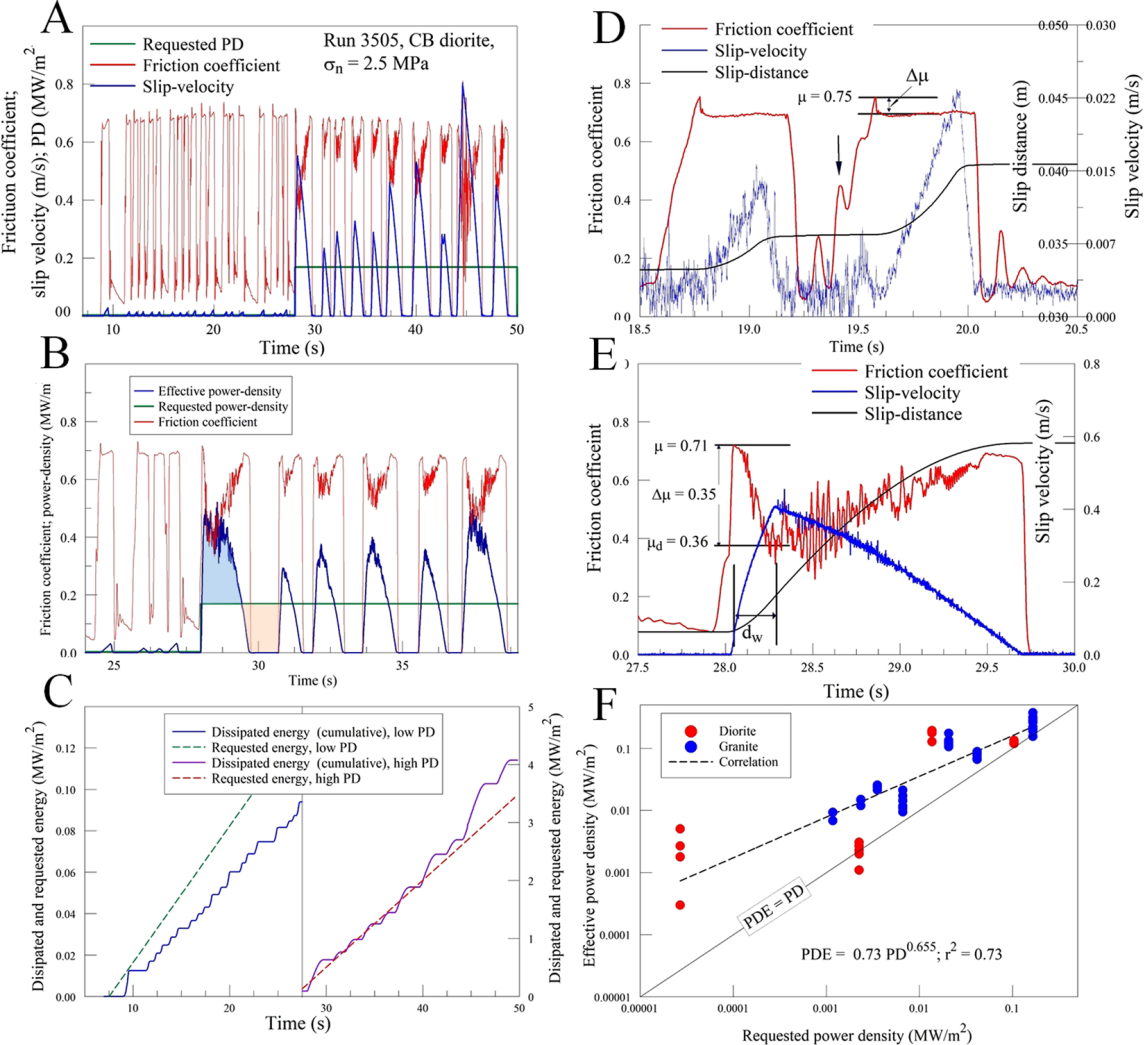
Observations of event-motion experiments (see text). (**A**) Experiment 3505 with two levels of requested PD (green), displaying the friction coefficient (red) and slip-velocity (blue). (**B**) Evolution of requested (PD) and effective (PDE) power-densities for part of the experiment (24–39 s); note: light blue area for PDE > PD, and light orange area for PDE < PD. (**C**) The integrated power-densities with respect to time for both requested PD (dashed lines) and measured PDE (solid curves); integration at 256 samples per s; note the different scales for low and high PD. (**D**) Two creep events of the low PD stage; note a wavy, gradual rise of the shear stress (red curve), with temporary drops of the rising shear stress (black arrow); slip ceased at t = 20.03 s leading to drop of the shear-stress; total slip-distance is 4.9 mm (black curve). (**E**) A microseismic event that slipped during the high PD stage of experiment 3505. Slip initiated at peak friction, μ_S_ = 0.71, and reached V = 0.49 m/s at acceleration of 2.1 m/s^2^. The event lasted 1.64 s with total slip-distance of 0.56 m (black curve). F. Comparison between PD and the integrated PDE during the 61 selected events that cover the full range of requested PD (see text).

Unlike the good fit between the PDE and PD curves for the entire duration of the stable-motion runs (Fig. 2), the event-motion style displays different relationships that are discusses for experiment #3505 (Fig. 3A–C). In this experiment, a diorite sample that was sheared under σ_n_ = 2.6 MPa was subjected to two levels of requested PD (low PD = 0.0066 MW/m^2^ followed by high PD = 0.165 MW/m^2^). Figure 3A displays the complex time evolution of the slip-velocity (blue), and the measured ratio μ  = [shear stress]/[normal stress] (red); for simplicity, μ is termed ‘friction coefficient’. Figure 3B displays the requested power-density (green), the effective power-density (blue), and the friction coefficient. Here, the PDE curve does not fit the PD curve; for example, during the period of 28–29.4 s, the PDE is larger than PD (light blue area in Fig. 3B), and inverse occurs during the 29.4–30.7 s, period (light orange area in Fig. 3B). To explore this misfit, Fig. 3C displays the integrated power-densities for both PD (dashed lines) and PDE (solid curves) with respect to time (integration at sampling rate of 256 s^−1^). An integrated power-density is the dissipated energy per area with MJ/m^2^ units. The integrated PDE curves exhibit general linear trend with temporal steps (Fig. 3C), in which the linear trend indicates the time-average of PDE that fits the constant PD (dashed line in Fig. 3C), and the temporal steps indicate the multiple events; these events are discussed in detail below. Note that the integrated PDE curve of the of the first part of the experiment is about 25% below the integrated low PD, whereas the integrated PDE curve of the of the second part is in good agreement with the integrated high PD. We attribute the mismatch of the first part to the limitation of the power control.

Experiment, #3505, exhibits several noticeable features:

1. A sequence of multiple events, 18 small slip events during the low PD (0.0066 MW/m^2^) and 10 large slip events during high PD (0.165 MW/m^2^).
2. The static friction coefficient is 0.72 ± 0.05 for all events.
3. The events display an abrupt rise and drop of the friction coefficient.

#### Creep events and stick-slip events

We now examine in detail two typical slip events of experiment #3505. The event (Fig. 3D) under PD = 0.0066 MW/m^2^, lasted less than a second (t = 19.25–20.03 s), it began with a wavy, gradual rise of the shear stress (red curve), and the temporary drops of the rising shear stress (black arrow), which were associated with small velocity pulses (blue curve). The main slip initiates at peak friction, μ = 0.75, and slip accelerates gradually to V = 0.023 m/s. During slip acceleration, the friction drops by Δμ = 0.054 to a constant level. As slip ceased (t = 20.03), the shear stress-drops as the apparatus relaxed. The total slip distance was 4.9 mm. This event displays a typical evolution for events at low PD that are characterized by gradual shear stress build-up, small friction drops, relatively small slip-velocity, and short slip distances. Due to these low intensity properties, we regard these events as creep events.

The second selected event of experiment #3505 displays a distinctly different evolution (Fig. 3E). Here, the requested power-density is high (0.165 MW/m^2^), the event lasted 1.64 s (t = 28.02–29.66 s), and displays an abrupt rise of the shear stress (red curve). Slip initiated at peak friction, μ = 0.71, and reached V = 0.49 m/s by acceleration of 2.1 m/s^2^. During slip acceleration, the friction drops by Δμ = 0.35 to a minimum and started rising as the slip decelerated. The fault stopped after slipping 0.56 m (black curve), and the shear-stress dropped as the apparatus relaxed. The event of Fig. 3E displays typical evolution for high PD events that are characterized by abrupt rise of shear stress, significant friction drops, large slip-velocity, and long slip displacements.

The exhibited intense acceleration and associated weakening that were followed by deceleration and strength recovery of these slip events (e.g., Fig. 3A,E), as well as the large slip velocities (up to 0.8 m/s) and displacements (up to 0.5 m) are similar in intensity to the equivalent parameters of moderate earthquakes (M_w_ = 3~5). Further, this behavior of weakening followed by strengthening is typical of the slip-pulse style of earthquake rupture^13^. We interpret these slip events as unstable, stick-slips that are experimental analogues of natural earthquakes. We further envision that the constant requested PD (green in Fig. 3A, and dashed lines in Fig. 3C) is analogous to the constant tectonic loading, and that during the unstable events the dissipated energy rate (PDE) is larger than the constant energy rate (PD) (light blue area in Fig. 3B). The inverse energy-flux relationship is manifested by periods of fault lock (light orange area in Fig. 3B) that are analogous to natural interseismic periods. The following analysis focuses on the unstable events.

## Power-Density Control of Fault Weakening and Slip-Velocity

We conducted 39 experimental runs with Radiant Red granite (RRG) and 37 runs with Charcoal Black diorite (CBD)^13,16^. The requested power-density ranged over more than three orders of magnitude (PD = 0.000027–0.17 MW/m^2^), and to analyze to full range, we selected a database of 42 and 19 slip events for CBD and RRG, respectively, with equal number of events for each level of requested PD (Table S1). We integrated the PDE for each of these 61 events and compared these values to the requested PD (Fig. 3F). This plot indicates, as expected, that PDE during an event is always larger than long term PD (above, Fig. 3B), and that PDE increases with increasing PD with power relations of PDE = 0.73·PD^0.655^ (r^2^ = 0.73) (Fig. 3F). We find that although the experiments are driven by the power-control system (Fig. 2A), the mechanical behavior of the experimental faults reflect on the actual, effective power-density. Therefore, the following quantitative analysis is based on the effective power-density.

First, the slip events display acceleration-deceleration stages without reaching a steady-state velocity, and the measured friction coefficients display corresponding evolution (Fig. 3A,D,E). Figure 3D,E displays the present definitions of friction drop, Δμ = μ_s_ − μ_d_, where μ_s_ and μ_d_ are the static and dynamic friction coefficients, respectively, and D_W_ is the weakening displacement. The dynamic friction coefficient, μ_d_, also shows a systematic weakening as function of maximum slip-velocity in a trend that agrees well with weakening trends in previous studies^13,17–19^. This agreement suggests similar weakening mechanisms at the micro-scale.

**Figure 4:**
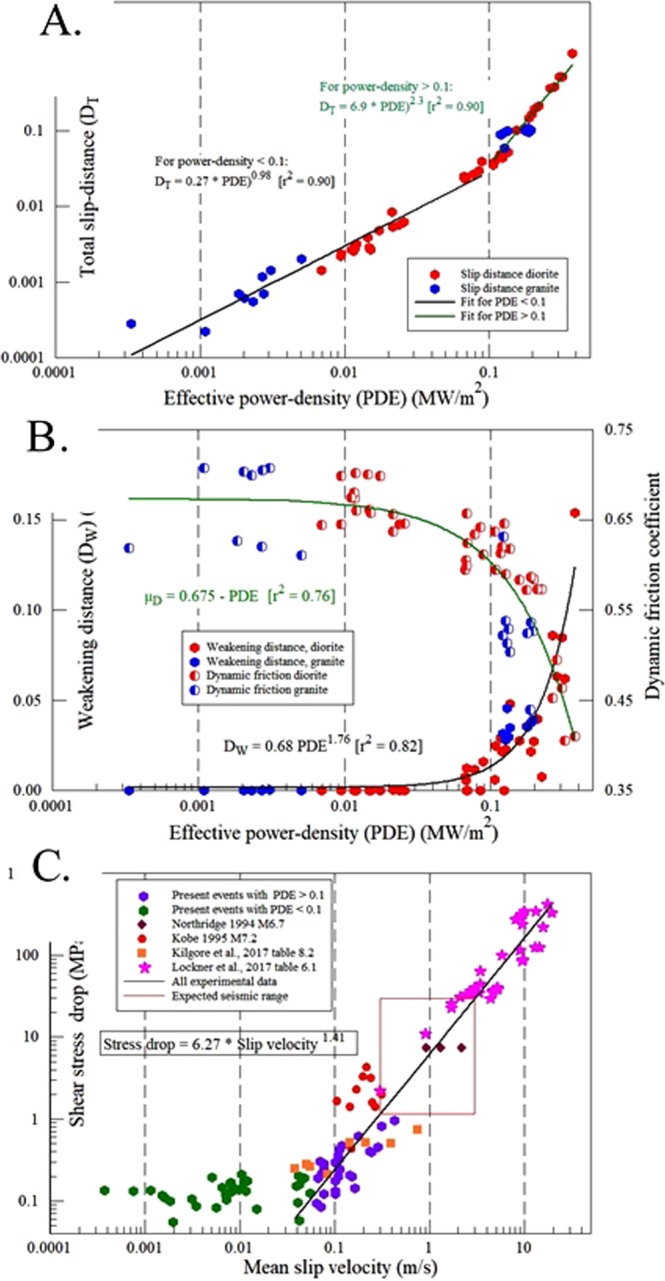
Slip characteristics of 61 unstable events as a function of the effective power-density. (**A**) Total slip-distance versus effective power-density. Note the slope transition at PDE = 0.1 MW/m^2^. (**B**) Dynamic friction coefficient, μ_d_, and weakening slip-distance, D_W_, (defined in Fig. 3B) versus PDE. Note change of slopes at PDE ≈ 0.1 MW/m^2^. (C) Relationship between stress-drop, Δτ, and associated mean slip-velocity, V_slip_, for (see legend): present experimental events [separated to PDE < 0.1 (green), and PDE > 0.1 (purple)], published experimental data^16,21^, seismic data of Kobe earthquake^25^, and Northridge earthquake^26^. The red rectangle bounds the expected range for seismic data based on the experimental results. Details in text.

Next, we examine the relationships between the events’ characteristics and the effective power-density (PDE) by plotting the measured parameters of total slip-distance, D_T_ (Fig. 4A), dynamic friction coefficient, μ_d_, and the weakening slip-distance D_W_ (Fig. 4B) (these parameters are defined in Fig. 3D,E). The total slip-distance reveals two zones: slip-distance of events with PDE < 0.1 MW/m^2^ have a power-law slope of 0.98 relatively to PDE, and events with PDE > 0.1 MW/m^2^ has a power-law slope of 2.3 (Fig. 3A). The non-linear relationships of dynamic friction coefficient, μ_D_, and the weakening slip-distance, D_W_, also show a distinct slope change at PDE ≈ 0.1 MW/m^2^ (Fig. 3B).

Following the observations of two PDE zones in the slip-distance plot (Fig. 4A) and the curves in the weakening plot (Fig. 4B), we divide the events into two groups, one of PDE < 0.1 and the second of PDE > 0.1. The two groups display a clear difference with respect to the experimental stress-drop, Δτ (Fig. 4C): the PDE < 0.1 events yield small stress-drops that are independent of the slip-velocity over two orders of magnitude, whereas the PDE > 0.1 events display distinct dependence of the stress-drop on slip-velocity.

## Discussion

### Relationship between stress-drop and slip-velocity

We examine the generality of the relationship between stress-drop and slip-velocity (Fig. 4C) by incorporating in the plot two sets of experimental data: (A) 37 shear experiments along saw-cut samples in a triaxial cell over a confining pressure range of 40–400 MPa^20^ (Table 6.1 in 20); and (B) eight shear experiments in a direct shear configuration at a normal stress of 2 MPa^21^ (Table 8.2 in 21). These two series in triaxial and direct shear are complementary to the presented rotary shear data in loading style and expand the range of observations to more than three orders of magnitude. The three experimental sets reveal a systematic relationship of Δτ  = 6.27·(V_slip_)^1.41^ (r^2^ = 0.95), where Δτ is in MPa, and V_slip_ is the mean velocity in m/s. We further incorporated in Fig. 4C data from two earthquakes^19,22^. Ide & Takeo (1997)^19^ analyzed the 1995 M7.2 Kobe earthquake and calculated the local slip-velocity, slip-distance and stress-drop at ten fault segments (depth 2.5 to 17 km, figs 4 and 8 in 19). Wald *et al*.^22^ calculated the slip history of the 1994 M6.7 Northridge earthquake from strong-motion, teleseismic, GPS, and leveling data, and the mean results for the entire fault are listed in Table 5 of ref.^23^. The plot of the data of these two earthquakes (Fig. 4C) fit the trend of stress-drop to slip-velocity as determined in the three experimental series. Further, this trend fits a wider range of seismic data, schematically represented by the red rectangle in Fig. 4C, which is bounded by 0.3–3.0 m/s range of slip velocities during 15 moderate earthquakes^24^, and stress drop of 1–10 MPa for moderate and large earthquakes^25^.

The relationship between stress-drop and slip-velocity are similar for experiments and earthquakes for wide range of settings and over three orders of magnitude (Fig. 4C). While we can quantify the power-density only for the present experiments, we propose, based on the similarity in Fig. 4C, that these slip events are controlled by the energy-flux to the experimental and natural faults. Under low power-density (green dot in Fig. 4C), the slip-velocity and stress-drop are low, typically for creep events. In contrast, high power-density induces high slip-velocity that leads to intense stress-drops. Below, and in the Supplementary Information section, we examine the possible application of the stress-drop/slip-velocity relations to crustal earthquakes.

### Energy-flux control of earthquake frictional slip

We show above that the energy-flux controls the style of fault slip in experiments (Figs 2, 3) and that the experimental relationship of stress-drop and slip-velocity fits the equivalent in moderate earthquakes (Fig. 4C). We now argue that energy-flux in crustal blocks may control the slip style along natural faults and the slip-velocity during earthquakes. A model for the energy-flux control on a crustal scale is derived for a long, vertical strike-slip fault that is loaded by a deep, underlying dislocation (Fig. 1A,B); the model details are presented in the Supplementary Information. During the interseismic period, the crust deforms within an energy-storage-belt of width W, in which the deforming crust has a shear modulus, G, and a shear wave speed V_S_. (Fig. 1A). As shown above (Eq. 1), the energy-flux, E_F_, which is equal to the frictional dissipation rate (PD), is the product of dynamic frictional strength and slip-velocity. By rearranging Eq. (1) for a fault with depth average frictional resistance, S_F_, the slip-velocity V_slip_ is

2 $${{\rm{V}}}_{{\rm{s}}{\rm{l}}{\rm{i}}{\rm{p}}}={{\rm{E}}}_{{\rm{F}}}/{{\rm{S}}}_{{\rm{F}}}$$

where E_F_ is the energy-flux during the earthquake. The rate of elastic energy flow in crustal blocks is bounded by the shear wave velocity, VS, and accordingly, the energy-flux from the storage belt to the fault is bounded by V_s_. Thus, the fault slip-velocity, V_slip_, during an earthquake is a function of the dynamic frictional strength, the shear wave velocity and the width of energy-storage-belt (Supplementary Information). Application of this relation to known geodetic and *in-situ* properties of the San Andreas fault^10,26–29^, indicate that the slip-velocity during large earthquakes is bounded to 1–3 m/s, in agreement with seismic observations^24,25^ (Supplementary Information).

## Conclusions

We propose that the similarity of the slip-velocity and stress-drop relationship in our experiments and moderate earthquakes stems from similarity of the non-trivial history of slip-velocity and shear-stress. The experimental events display accelerated loading, abrupt weakening and strengthening (Fig. 3), and it is expected that earthquakes would have similar complex velocity evolution^5,30^ without reaching a steady-state stage^13^. This realization that laboratory simulation of large earthquakes requires simulation of the complex slip histories in natural earthquakes, led to experiments with direct input of non-trivial velocity history^13,23,31^, and experiments with finite supply of energy^32^. The present analysis extended these approaches in two central aspects: power-density control that generates spontaneous complex events that are comparable to slip during moderate earthquakes, and balancing energy and energy-flow on a crustal scale.

## Methods

### Apparatus

**Figure 5:**
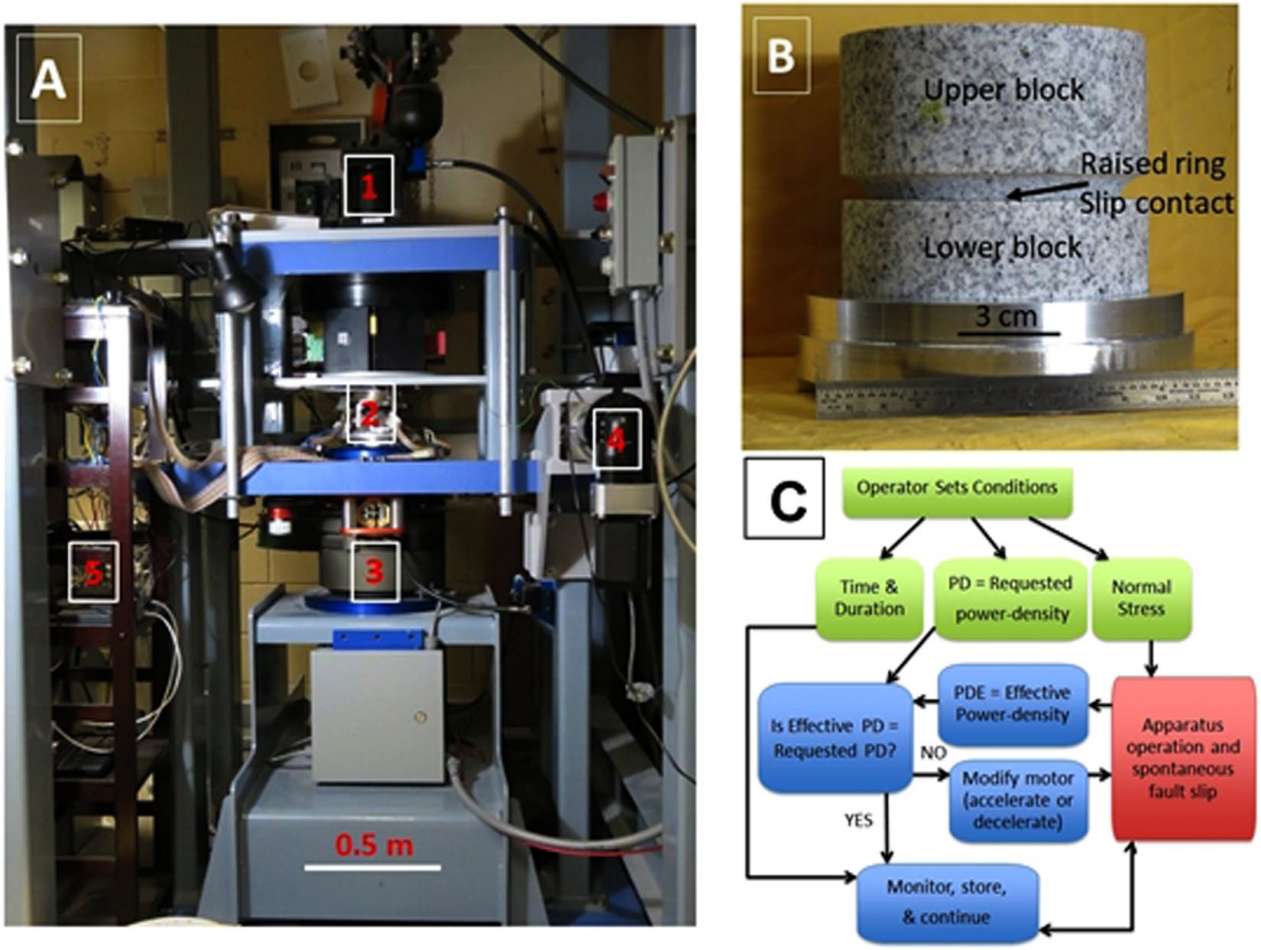
The rotary shear apparatus^19^. (**A**) General view showing: 1. Axial load piston; 2. Sheared sample position; 3. Electromagnetic clutch connection to high-velocity motor; 4. Stepping motor for low-velocity shear; and 5. High-speed monitoring system. (**B**) Sample blocks made of Sierra-White granite showing the contact ring between rotating lower block and upper block.

The experiments were conducted on the high-speed, rotary shear apparatus (ROGA) at the University of Oklahoma^21^ (Fig. 5). The apparatus can apply unlimited shear along rock blocks at slip-velocities up to 2 m/s and normal stresses up to 30 MPa. The apparatus frame is 1.8 m tall with two decks that are connected to each other by four internally reinforced legs. The sample is placed between the two decks, and it is loaded by the rotary power train from below and by the normal stress from above. The power system includes: (1) A 100 HP three-phase Baldor electric motor, and a controller that provides constant torque of up to 3,000 Nm from 0 RPM to 3300 RPM. The shaft is powered by the motor with 1:6 velocity reduction sprockets; (2) A 225 kg flywheel to boost the motor torque for short rise time during high-speed tests; (3) An electro- magnetic large clutch (Ogura) that is capable of full engagement in 30 ms; and (4) A hydraulic piston system (Enerpac) with axial load up to 9,500 N. The control and monitoring system includes National Instruments SCXI-1100 with modules 1124 (analog control) 1161 (relay control), 1520 (load cell/strain gauge), and 1600 (data acquisition and multiplexer), as well as a USB-6210 (encoder measurements). Digital sampling rates of up to 10 kHz are available. Load-cells for axial load and torque (Honeywell), gouge dilation/compaction is measured with four eddy-current sensors (Lion Precision) (1- micron accuracy), temperature measurement is with thermo-couples (Omega), and sample radial velocity encoder.

### Power-density control

The power-density control in our system is performed with a dedicated LabView program that utilizes the ‘proportional–integral–derivative’ (PID) controller of the apparatus motor (Fig. 5C). Before running an experiment, the operator selects time, history of the requested PD, and the experimental settings (normal stress etc.) (green squares in Fig. 5C). The responses of the experimental fault and the motor controller that are monitored continuously by the National Instruments system (red square), including the data of shear stress (strain-gauge sensor) and slip-velocity data (motor) as digital voltage values. The program uses these values to calculate the ‘PDE = Effective power density’ (blue squre), and compared it to the requested PD (blue square ‘Is effective PD = Requested PD?’). Depending on this comparison (No/Yes arrows), the motor velocity is modified or not (relevant blue squares).

This PD control procedure is adjusted by tuning the PID parameters before running, and once the test starts, the interconnected system control and fault behavior are spontaneous. Running under PD control requires setting the following: the controlling Labview algorithm (Fig. 5C) for the relative contribution of slip-velocity and shear stress, a set of 6–7 parameters of the motor controller PID, the maximum motor speed, and the rate of control and sampling. The system capacity for combined control and computation is 256 samples/sec, and the accuracy of the control is limited by this sampling rate.

We found that the slip style strongly depends the intensity of the nominal PD. First, we found that the stable-motion (Fig. 2) is associated with low PD, low requested maximum velocity, and low sampling rates; which together allow for matching of PD and PDE. We found that medium to high PD generated event-motion, as seen, for example, in experiment #3505 with two levels of PD while keeping all other parameters constant. The experimental record of this experiment (Fig. 3) displays different slip styles that vary with the intensity of the temporal requested PD. As discussed in the text, the experimental results show matching PD and PDE for the stable-motion (Fig. 2), agreement between integrated PD and integrated PDE for the event-motion runs (Fig. 3B), and general agreement with the integrated PDE of isolated events and the requested PD (Fig. 3C).

Most important, we are interested in revealing the effect of energy-flux (=power-density) on fault slip, and as the fault slips by the effects of the effective-power-density (PDE), the analysis of the slip events in the event-motion cases (Fig. 4) is based only on the PDE values.

### Tested samples

The rock types used in the experiment have the commercial names of Radiant Red granite (RRG) and Charcoal Black diorite (CBD)^18^. RRG was quarried in Fredricksburg, TX, it’s bulk density is 2614 kg/m^3^ and a uniaxial strength of 146.5 MPa. The powder X-ray diffraction (XRD) composition includes quartz (43.6%), albite (19.7%), microcline (22.8%), and biotite (13.9%) in weight percent. CBD was quarried in St. Cloud, Minnesota, it’s bulk density is 2,723 kg/m^3^ and uniaxial strength of 173.9 MPa. Powder XRD analysis shows quartz (16.7%), albite (32.7%), microcline (26.8%), amphibole (7.2%), and biotite (16.7%), in weight percent.

Each sample includes two cylindrical blocks, diameter = 101.6 mm, height = 50.8 mm. The upper block has a raised ring with ID = 63.2 mm and OD = 82.3 mm (Fig. S1), and the two blocks are pressed across this raised ring. This ring-shape design has an advantage of generating approximately uniform slip-velocity, and the small diameter difference minimizes the linear velocity difference at ring edges of the sample. The fault surfaces were ground flat and roughened with #400 powder.

## Supplementary information

Supplementary information
