# Supplementary material for: Energy-flux control of the steady-state, creep, and dynamic slip modes of faults
Source: Sci Rep. 2019 Jul 23;9:10627. doi: 10.1038/s41598-019-46922-1 (PMC6650398; doi:10.1038/s41598-019-46922-1)
Supplement: Supplementary file 1 — Supplementary information [file 41598_2019_46922_MOESM1_ESM.pdf]

Supplementary information

Energy-flux control of the steady-state, creep, and dynamic slip modes of faults

Authors: Ze'ev Reches*, Ximeng Zu, Brett M. Carpenter

MODEL OF ENERGY-FLUX CONTROL OF EARTHQUAKE FRICTIONAL SLIP

We experimentally demonstrated that energy-flux controls the slip style along experimental faults (text), and proposed that this control may also affect natural earthquakes as outlined in equation (2) and Fig. 4C. Here, we derive a simple model that examines relationships between energy-flux and earthquake slip along a crustal fault by simultaneous balancing two energy parameters: (A) a static balance between the total available elastic energy and the total energy dissipated along the fault; and (B) a dynamic balance between energy-flux to the fault and energy-dissipation-rate along the fault.

The model considers a long, vertical strike-slip fault that is loaded by a deep, underlying dislocation (Figs. S1A, B). The fault is locked during the interseismic period, its depth average frictional resistance is S_F, and the surrounding crust has a shear modulus, G, and a shear wave speed V_S. During the interseismic period the slip along the deep dislocation deforms the crust within an <u>energy-storage-belt</u> of width W, on both sides of the locked fault (Fig. S1A). For simplification, we assume uniform elastic strain within the energy-storage-belt, W; in the model application to the San Andreas fault (below), this assumption of strain distribution is reexamined. Defining ε_S as the elastic shear strain available for earthquake slip within this belt, then the available elastic energy per unit area of the fault is

$$E_E = 0.5 \cdot \varepsilon_S^2 \cdot G \cdot W \qquad \text{(S1)}$$

We consider here only the frictional dissipation, which is largest component of earthquake dissipation[1,2]. The available elastic energy, E_E, in the energy-storage-belt equals the energy dissipated by the seismic slip distance, d. along the fault section with dynamic frictional strength of S_F, thus

$$E_E = d \cdot S_F. \qquad \text{(S2)}$$

As discussed in the text, the power-density needed for the frictional dissipation during slip at velocity, V_{slip}, is the product of dynamic frictional strength and slip-velocity, with units of MW/m^2,

$$PD = S_F \cdot V_{slip}. \qquad (S3)$$

If the elastic energy flow rate in the host blocks equals the shear wave velocity, V_S, then the average time needed for the elastic energy to reach the fault from the center of the energy-storage-belt is

$$t = (W/4) / V_S. \qquad (S4)$$

Thus, the energy-flux of the available elastic energy from the center of the storage belt to the fault is the ratio of total available elastic energy (S1) and the time bounded by the shear wave velocity (S4),

$$PD_A = 2 \cdot \varepsilon_S^2 \cdot G \cdot V_S \qquad (S5)$$

We regard PD_A as the 'available power-density' as it reflects the elastic power available to drain the entire width of the interseismic stored energy ('deformed zone' in Fig. S1A). Equating the available power, PD_A, and the dissipation power, PD, [equations (S3) and (S5)] provides the condition for which slip can occur during an earthquake without energy starvation,

$$V_{slip} = 2 \cdot \varepsilon_S^2 \cdot G \cdot V_S / S_F \qquad (S6)$$

This equation indicates that the derived slip velocity, V_{slip}, is proportional to the total available elastic energy ($0.5 \cdot \varepsilon_S^2 \cdot G$), with a reciprocal relationship to the fault's dynamic strength, S_F. The model slip-velocity is plotted in Fig. S2A as a function of the mean S_F for a range of elastic shear strains (ε_S). The model results (Fig. S2) are derived from "typical" crustal values of G = 50 GPa and V_S = 3 km/s. The gray zone in Fig. S2A marks the 0.5-3.0 m/s range of slip velocities during 15 earthquakes in the range of Mw = 5.6-7.2[23]. The model results indicate that slipping at this velocity range requires elastic strain ε_S of $1\text{-}4 \cdot 10^{-4}$ in the crust for slip along a fault with mean dynamic strength up to 25 MPa; these values will be explored below. The model refers to the mean values of V_{slip} and S_F, and it does not consider the time evolution of slip-velocity during seismic slip.

Interestingly, the derived slip-velocity in (S6) does not depend on W, the width of the energy-storage-belt. This feature indicates that the intensity of the available elastic strain controls both the slip-displacement, d (equations S1, S2), and the slip-velocity (S6). A fault with a narrow energy-storage-belt, could slip at high velocity ($v > 0.5$ m/s) if the intensity of the elastic strain is high enough. At the same time the slip-displacement would be short as indicated by equating (S1) and (S2) which shows that $d = (0.5 \cdot \varepsilon_S^2 \cdot G/S_F) \cdot W$.

ENERGY-FLUX AS SLIP-VELOCITY CONTROL

The present model is based on simultaneously balancing static energy and dynamic energy-flux, and predicts the frictional slip-velocity, V_{slip}, as a function of the dynamic frictional strength and the interseismic shear strain in the crustal blocks (Fig. S2A). We now apply this model to the segment of the San Andreas fault, south of Parkfield. The geodetic surface velocity in this region was analyzed by Maurer & Johnson (2013)[10], and their profile C-C' (their Fig. 2) is displayed here (blue dots in Fig. S2B). It was already noted[10] that this velocity profile fits well the surface velocity distribution above a vertical, deep seated, strike-slip dislocation that is overlain by a locked fault; the configuration is shown in Fig. 1A. We used the classical dislocation relationships[3, 33] to show that the geodetic data (blue dots, Fig. S2B) fit a slip-velocity of 34 mm/y along a vertical strike-slip dislocation with its tip at 20 km depth (blue curve, Fig. S2B). For an interseismic period of 130 years[34], the cumulative displacement along the same dislocation is 4.42 m, and we used this value to calculate the surface shear strain in an energy-storage-belt of $x = \pm 80$ km (red curve, Fig. S2B). The calculated maximum shear strain is $\varepsilon_S = 3.54 \cdot 10^{-4}$ at $x = 0$, that drops to half the maximum ($\varepsilon_S = 1.77 \cdot 10^{-4}$) at $x = \pm 20$ km (dashed red curve in Fig. S2B). The expected slip velocity along this fault segment due to energy-flux is calculated by substituting these two strain values and dynamic frictional strength range of $S_F = 15\text{-}25$ MPa into equation (S6). As a final

point, these calculations revealed a slip-velocity range of 0.37-2.4 m/s (blue region in Fig. S2B) that fits both the slip-velocity range of earthquakes (gray zone in Fig. S2A), and the estimated dynamic strength for the San Andreas fault in this region, which is on the order of 10-20 MPa based on the analyses of heat flow[25-27] and in-situ stress fields[28].

References

33. Weertman, J. & J. R. Weertman, *Elementary Dislocation Theory*, 213 pp., Macmillan, New York (1964).

34. Biasi, G. P., Weldon, R. J., Fumal, T. E., & Seitz, G. G. Paleoseismic event dating and the conditional probability of large earthquakes on the southern San Andreas fault, California. *Bull. Seismological So. Am.*, **92**, 2761-2781 (2002).

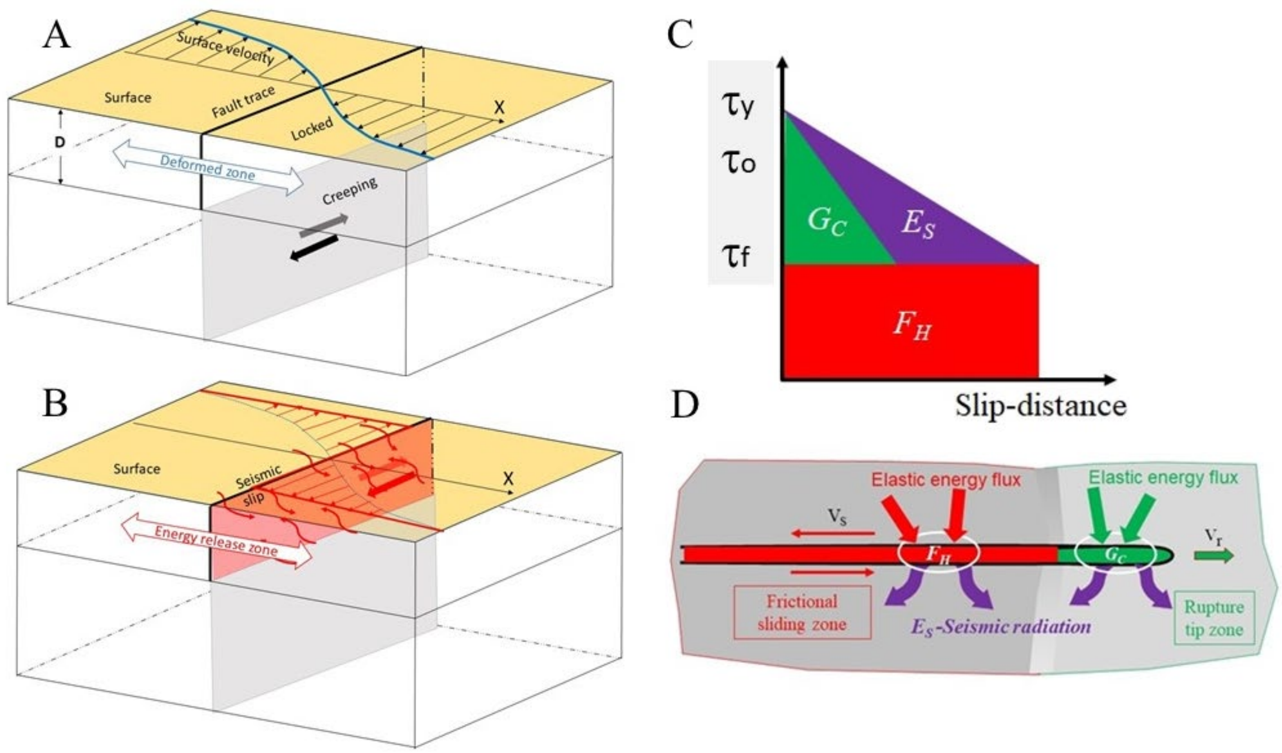

***Fig. S1**. (same as Fig. 1 in text). A. B. Schematic view of an earthquake cycle along a large strike-slip fault. During the interseismic period (A), slip occurs along the deep part while elastically deforming (blue curve) the upper layer. During the earthquake (B), the locked fault slips unstably (pink zone) facilitated by energy-flux (red, wiggly arrows) from the energy release zone. C. Energy dissipating processes[1, 2] during an earthquake as function of slip distance. G_C–fracture energy; F_H-frictional energy; E_S- radiated, seismic energy. D. Energy-flux toward the two general zones of energy dissipation: the rupture tip (green) and the frictional sliding block (red) in the wake of the earthquake rupture tip.*

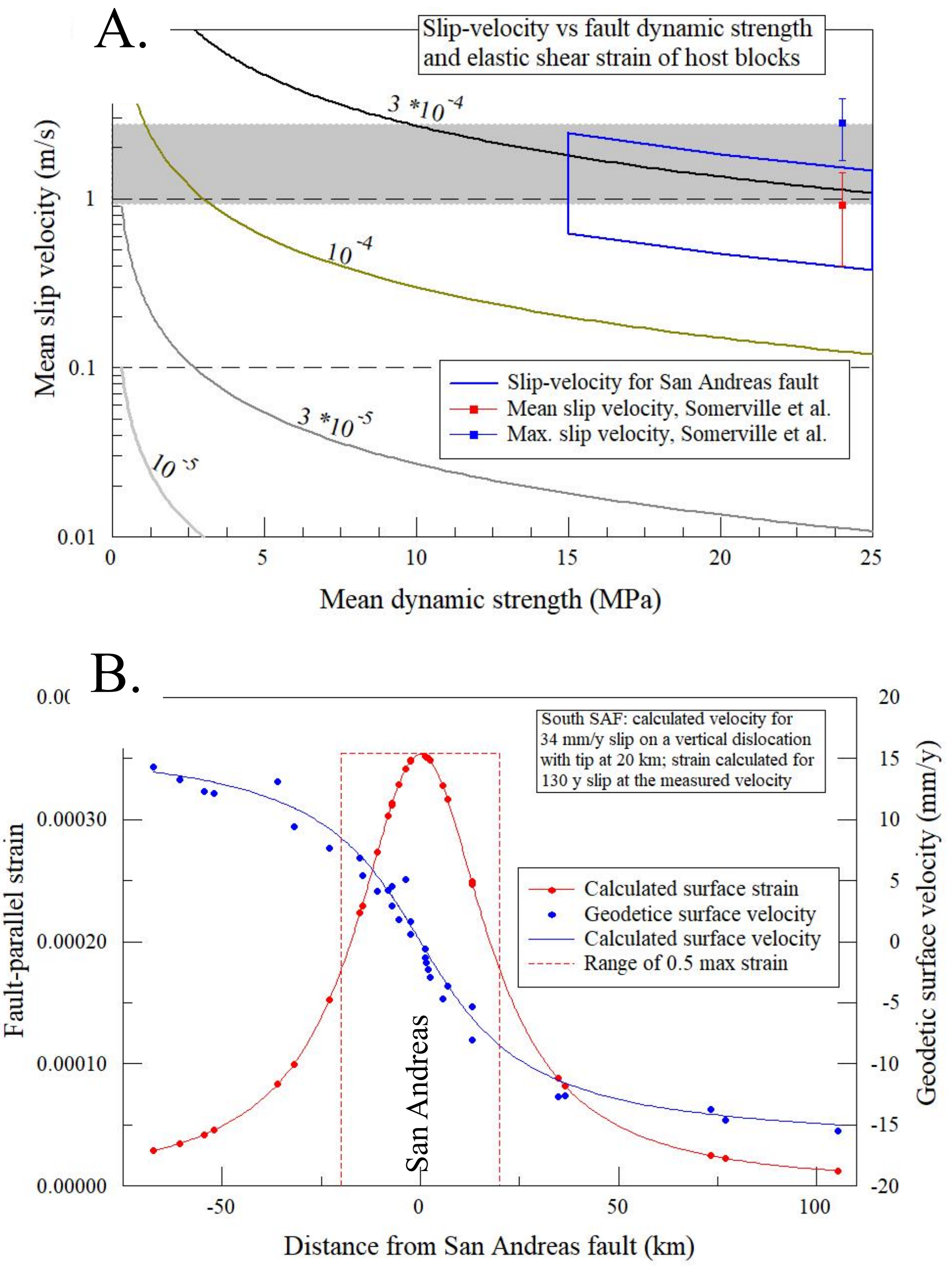

Fig. S2. *A. Calculated slip-velocity (equation S6) for frictional sliding along a fault with dynamic frictional strength (S_F horizontal axis), and released elastic energy (marked curved with fault-parallel shear strain intensity. Calculated for crust of G = 50 GPa and V_S = 3 km/s (text and equation 6). Gray area – range of earthquake slip-velocity of 15 earthquakes[23] bounded by the average value (red symbol) and maximum value (blue symbol); blue quadlateral – expected slip-velocity for the southern San*

Andreas fault according to the energy-flux model (see text). B. Fault-parallel velocity and associated shear strain across the southern San Andreas fault (see text). Blue dots – geodetic surface velocity[10]; blue curve – dislocation velocity fit to the to the geodetic velocity for a strike-slip dislocation with tip at 20 km depth and slip-velocity of 34 mm/y; red curve with dots – surface shear strain calculated for the dislocation with displacement of 4.42 at 20 km depth; dashed red rectangle – bounding the region between the maximum and one half of the shear strain.

Table S1. The experimental data for the analyzed events, including requested PD, effective power-density, PDE, normal stress, static shear stress, shear stress-drop, friction coefficient.

Test No.	PD	PDE	σn	Static τ	Δτ	μ static	Δμ	Slip-velocity		Slip distance		Rise time
	MW/m^2	MW/m^2	MPa	MPa	MPa			mm/s		mm		sec
								Max.	Mean	Total	Weak	
3505 CBD (small)	0.0066	0.0214	2.64	1.80	0.08	0.686	0.030	19.2	14.9	8.5	0.0	0.57
	0.0066	0.0117	2.64	1.92	0.15	0.731	0.056	9.6	8.3	3.1	0.0	0.37
	0.0066	0.0116	2.63	1.91	0.13	0.728	0.048	9.6	8.2	2.6	0.0	0.32
	0.0066	0.0111	2.62	1.92	0.16	0.732	0.058	9.6	7.8	2.7	0.0	0.34
	0.0066	0.0145	2.59	1.95	0.14	0.754	0.053	16.8	9.6	3.9	0.0	0.41
	0.0066	0.0095	2.59	1.96	0.15	0.754	0.055	9.6	6.5	2.3	0.0	0.36
	0.0066	0.0174	2.59	1.94	0.13	0.750	0.052	16.8	11.6	4.8	0.0	0.42
	0.0066	0.0118	2.59	1.99	0.17	0.766	0.064	9.6	8.0	2.8	0.1	0.35
4050 CBD	0.00117	0.0094	3.37	2.37	0.19	0.702	0.056	6.0	5.1	2.2	0.0	0.44
	0.00117	0.0069	3.37	2.26	0.09	0.670	0.026	4.8	3.5	1.4	0.1	0.41
	0.00234	0.0120	3.37	2.31	0.08	0.685	0.025	12.0	5.7	3.2	0.1	0.56
	0.00234	0.0148	3.37	2.35	0.12	0.695	0.034	10.8	7.1	2.9	0.2	0.41
	0.00234	0.0151	3.38	2.32	0.10	0.688	0.030	12.0	7.2	2.7	0.2	0.38
	0.00351	0.0238	3.38	2.35	0.17	0.693	0.048	18.0	11.0	5.8	0.2	0.52
	0.00351	0.0257	3.39	2.37	0.18	0.698	0.052	21.5	12.0	6.2	0.2	0.52
	0.00351	0.0215	3.39	2.38	0.21	0.700	0.062	16.8	10.3	5.3	48.1	0.52
3490 CBD	0.0205	0.1238	2.71	1.82	0.08	0.678	0.032	134.1	71.3	44.0	29.2	0.62
	0.0205	0.1354	2.73	1.79	0.12	0.657	0.039	143.7	75.9	52.2	35.7	0.69
	0.0205	0.1169	2.66	1.93	0.30	0.722	0.109	131.8	69.6	43.5	23.0	0.63
	0.0205	0.1754	2.63	1.93	0.43	0.719	0.146	208.4	112.8	108.4	21.6	0.96
	0.0205	0.1267	2.70	1.87	0.28	0.708	0.118	141.3	76.1	49.3	5.8	0.65
	0.0205	0.1197	2.64	1.85	0.22	0.696	0.075	136.5	72.9	48.4	24.6	0.66
	0.0205	0.1070	2.63	1.77	0.09	0.671	0.033	122.2	64.1	35.6	12.5	0.55
	0.0205	0.1077	2.63	1.76	0.19	0.663	0.068	124.6	66.7	38.3	11.6	0.57
3500 CBD	0.0415	0.0687	2.65	1.89	0.15	0.711	0.053	81.4	39.5	23.0	16.1	0.58
	0.0415	0.0776	2.65	1.87	0.19	0.701	0.067	88.6	46.1	26.5	7.6	0.57
	0.0415	0.0892	2.65	1.74	0.12	0.654	0.042	107.8	55.0	39.1	5.5	0.71
	0.0415	0.0666	2.66	1.64	0.06	0.616	0.021	83.8	41.9	24.7	0.0	0.59
	0.0415	0.0673	2.66	1.77	0.16	0.663	0.057	79.1	42.0	24.6	0.1	0.59
	0.0415	0.0681	2.67	1.80	0.20	0.674	0.075	79.1	41.1	24.7	0.2	0.60

	0.0415	0.0692	2.70	1.78	0.10	0.662	0.038	81.4	40.6	23.8	27.6	0.59
	0.0415	0.0851	2.72	1.92	0.19	0.714	0.072	88.6	47.3	29.9	27.4	0.63
3505 CBD (large)	0.165	0.1560	2.57	1.96	0.47	0.764	0.180	227.6	117.6	100.6	7.6	0.86
	0.165	0.1965	2.56	1.70	0.20	0.659	0.075	280.3	151.1	164.0	21.6	1.09
	0.165	0.2218	2.57	2.09	0.61	0.804	0.231	323.4	176.6	214.5	63.0	1.21
	0.165	0.1883	2.56	1.70	0.21	0.663	0.076	282.7	143.2	146.5	84.7	1.02
	0.165	0.2830	2.58	1.67	0.39	0.658	0.163	455.1	244.2	376.8	39.5	1.54
	0.165	0.3081	2.58	1.66	0.45	0.663	0.200	524.6	283.0	520.8	154.0	1.84
	0.165	0.2071	2.57	1.61	0.14	0.632	0.058	275.5	163.7	196.4	62.2	1.20
	0.165	0.3771	2.62	1.98	0.96	0.759	0.350	792.9	424.1	1061.8	85.8	2.50
	0.165	0.3194	2.57	1.87	0.81	0.720	0.315	543.8	308.9	518.9	0.0	1.68
	0.165	0.2649	2.58	1.59	0.40	0.632	0.180	443.2	236.8	360.8	0.2	1.52
3430 RRG	0.00224	0.0011	2.16	1.66	0.13	0.768	0.060	1.8	0.8	0.2	0.3	0.30
	0.00224	0.0031	2.16	1.56	0.02	0.718	0.010	4.8	2.2	1.4	0.1	0.66
	0.00224	0.0027	2.16	1.59	0.06	0.731	0.026	4.2	2.0	0.7	0.1	0.36
	0.00224	0.0020	2.16	1.63	0.11	0.754	0.051	3.0	1.6	0.6	28.2	0.39
	0.00224	0.0023	2.17	1.64	0.12	0.755	0.055	3.0	1.5	0.5	31.6	0.36
3435 RRG	0.1032	0.1256	2.19	1.58	0.28	0.719	0.129	220.9	105.3	93.4	31.4	0.89
	0.1032	0.1201	2.19	1.57	0.30	0.709	0.131	225.7	101.1	88.5	34.7	0.88
	0.1032	0.1219	2.19	1.43	0.12	0.652	0.055	230.4	100.4	89.8	29.5	0.89
	0.1032	0.1359	2.19	1.52	0.24	0.695	0.115	242.3	112.5	98.5	37.4	0.88
	0.1032	0.1322	2.19	1.60	0.32	0.729	0.149	242.3	110.8	99.6	39.2	0.90
3450 RRG	0.0136	0.1876	3.40	2.12	0.13	0.623	0.043	232.2	102.1	95.8	35.2	0.94
	0.0136	0.1948	3.39	2.27	0.35	0.664	0.104	244.2	108.3	101.5	44.3	0.94
	0.0136	0.1783	3.40	2.11	0.18	0.619	0.053	233.4	101.6	98.5	45.7	0.97
	0.0136	0.1866	3.40	2.03	0.20	0.594	0.060	251.4	108.3	108.3	0.1	1.00
	0.0136	0.1287	3.38	2.04	0.24	0.600	0.067	173.6	75.9	58.7	0.1	0.77
3452 RRG	0.0000268	0.0051	3.41	2.20	0.11	0.642	0.031	12.0	3.1	2.0	0.1	0.66
	0.0000268	0.0027	3.41	2.23	0.10	0.650	0.030	6.0	1.8	1.2	0.0	0.64
	0.0000268	0.0003	3.41	2.25	0.13	0.658	0.039	2.4	0.4	0.3	0.0	0.76
	0.0000268	0.0018	3.41	2.28	0.14	0.665	0.038	4.8	1.2	0.7	0.0	0.61
